# Supplementary material for: Towards women’s digital health equity: A qualitative inquiry into attitude and adoption of reproductive mHealth services in Bangladesh
Source: PLOS Digit Health. 2024 Oct 15;3(10):e0000637. doi: 10.1371/journal.pdig.0000637 (PMC11478865; doi:10.1371/journal.pdig.0000637)
Supplement: S1 File — (DOCX) [file pdig.0000637.s001.docx]

***Towards women’s digital health equity: A qualitative inquiry into attitude and adoption of reproductive mHealth services in Bangladesh***

*Transcription of responses from the IDIs [Anonymous]*

Contents

[Q0 2](#_Toc175162172)

[Q1 5](#_Toc175162173)

[Q2 8](#_Toc175162174)

[Q3 11](#_Toc175162175)

[Q4 14](#_Toc175162176)

[Q5 17](#_Toc175162177)

[Q6 20](#_Toc175162178)

[Q7 23](#_Toc175162179)

# Q0

| **Sl. No** | **Age** | **University** | **Married** | **Doctor for SRH** |
| --- | --- | --- | --- | --- |
| 1 | 25 | University of Dhaka | No | Yes |
| 2 | 24 | University of Dhaka | No | No |
| 3 | 24 | Bangladesh University of Professionals | No | No |
| 4 | 23 | University of Dhaka | Yes | No |
| 5 | 24 | University of Dhaka | No | Yes |
| 6 | 25 | Begum Rokeya University | No | No |
| 7 | 23 | Begum Rokeya University | No | Yes |
| 8 | 24 | Bangabandhu Sheikh Mujibur Rahman Science & Technology University | Yes | No |
| 9 | 23 | Bangabandhu Sheikh Mujibur Rahman Science & Technology University | No | No |
| 10 | 22 | Bangabandhu Sheikh Mujibur Rahman Science & Technology University | No | No |
| 11 | 24 | University of Dhaka | No | No |
| 12 | 24 | University of Dhaka | No | Yes |
| 13 | 24 | National university | Yes | No |
| 14 | 23 | National university | No | No |
| 15 | 23 | Bangladesh University of Professionals | Yes | No |
| 16 | 23 | Bangladesh University of Professionals | No | Yes |
| 17 | 24 | University of Dhaka | No | Yes |
| 18 | 24 | National University | No | Yes |
| 19 | 29 | University of Dhaka | Yes | No |
| 20 | 24 | Jahangirnagar University | No | No |
| 21 | 26 | Jahangirnagar University | No | No |
| 22 | 31 | Jahangirnagar University | Yes | No |
| 23 | 31 | University of Dhaka | Yes | Yes |
| 24 | 31 | University of Dhaka | Yes | No |
| 25 | 26 | Jagannath University | No | No |
| 26 | 26 | Jagannath University | No | No |

# Q1

| **Sl. No** | **B1. Would you please tell us about your feelings on reproductive mHealth services and its impact on the young people in Bangladesh?** |
| --- | --- |
| 1 | I think everyone should be aware of their reproductive functionality and health and if it comes in digital sources then that will be very helpful. |
| 2 | It's very useful because we don't have to remember the details rather it gives us necessary right information in time. |
| 3 | I do not believe I can explain properly or get proper or related information or solution through m health service. |
| 4 | The services are good, and I think these services have a great impact on young people. Young people require these services more. |
| 5 | At present, girls are more conscious about their reproductive health and sexual wellbeing. So, they use various apps for tracking their menstruation cycle, ovulation, if something is not right. They can control their eating habits for betterment. |
| 6 | I think it’s a very positive initiative. Youths in our country hesitate to talk about their reproductive health even if they’re facing difficulties. So, people can discuss about their problems without any awkwardness. |
| 7 | Good. |
| 8 | I think it's a unique idea & service. young people of Bangladesh will be known about personal health & they can use it for their any question or any information. |
| 9 | I think it's effective. However, it is important to have awareness and patience in fact-checking. |
| 10 | No idea regarding this matter. |
| 11 | It's important, as women in our country still feel the need to hide their complexities on their reproductive health. Breaking the taboo and normalizing, seeking help is a great first step. And services that are readily available on various virtual platform, makes it easier to access. |
| 12 | I think the apps are very user-friendly in cases of tracking period schedules and keeping up with health updates, such as weight gain/loss, ovulation etc. The younger people can get benefitted by them by easy tracking on period dates, keeping notes about changes they face every month etc. |
| 13 | Not used such type of mHealth services yet that’s why haven't enough knowledge regard these services. |
| 14 | It's very important now a days as the social media optimization. |
| 15 | It takes time to work efficiently with coverage of a large number of populations. |
| 16 | It’s very effective. |
| 17 | Actually, in today's world, due to extreme pollution and impurity in our food and unhealthy lifestyle our reproductive health is in a visibly vulnerable state. We girls talk about this and many of us use different apps to track period date. We try to gather knowledge from google, social media and using mobile health services while needed. Overall, my feeling about it is this is still not available and well known but people are getting serious about their reproductive health. So government and private sector should come forward to expand mHealth service and media outreach up to rural area too. |
| 18 | I think this is a great initiative as often it's uncomfortable for youngsters in Bangladesh to seek this kind of services in person. Because most doctors are judgmental and unsympathetic. |
| 19 | I haven’t taken any such service. |
| 20 | It’s important for our young generation. |
| 21 | I think mHealth service on reproductive issues has a great impact on the young people in Bangladesh as the you are very conservative in this country and sexual and reproduction related education is still acknowledged as taboo here. So mHealth services can be a useful method. But I will recommend a face-to-face health service. |
| 22 | It should be available. |
| 23 | It informs and will inform the future generation more about the appropriate health services. Eventually it will make people knowledgeable about health issues. |
| 24 | I think access to sexual and reproductive health service is extremely important for young people. It can ensure medical care and comprehensive sexuality education. Information regarding sexualities, sensuality and gender identities are very important these days. It can create impact on young people’s bodily autonomy and power over their lives. |
| 25 | It is an innovative idea. |
| 26 | Good sexual and reproductive health is important for women's general health and wellbeing. It is central to their ability to make choices and decisions about their lives, including when, or whether, to consider having children. |

# Q2

| **Sl. No** | **B2. What is your level of trust on mHealth as a reliable source of reproductive healthcare service in Bangladesh and why?** |
| --- | --- |
| 1 | I think the apps that are available on mobile phone are quite trustworthy. |
| 2 | Moderate level of trust as reproductive health is unpredictable. |
| 3 | Moderate. |
| 4 | Medium level of trust because sometimes they might provide misinformation. |
| 5 | Mobile health services are reliable now as medical science is advancing more. |
| 6 | It’s a 3/5 for me as I haven’t used it before, but the concept is great. |
| 7 | Medium level. |
| 8 | It's a new idea. As a beginner I trust this for the better. because of people of Bangladesh think that talking about personal health is a shameful thing. |
| 9 | Quite trustable. |
| 10 | Not aware about this. |
| 11 | It's decent. Since it works as a first base of knowledge on reproductive healthcare. And it's easily available. |
| 12 | They can be reliable as long as the data is protected. I trust the app I use as so far, I haven’t experienced any data breach. |
| 13 | Not enough trustworthy. |
| 14 | I never got any kind of mHealth service really don't know how worthy is the services. |
| 15 | I trust it will work but it will take time as well as most of our people are still unfamiliar to it due to various kind of limitations such as illiteracy, lack of campaign initiative or lack of equipment and so on. |
| 16 | I trust those sources because they provide effective information about reproductive health . |
| 17 | I personally trust mHealth service because people are now serious and due to social media, we know many specialists whom we see providing services. We Don't only relay on apps rather verify and google also. So, I can give 8 out of 10. |
| 18 | I think it can be trusted but not as much as clinics. As there are various tests and consultations involved to make proper diagnosis. |
| 19 | No idea. |
| 20 | If I rate the level of trust its 6/10 because doctors are corrupted and irresponsible in Bangladesh |
| 21 | Actually, I don’t have that much trust on this mHealth services because I don’t have any previous experience in this field and it’s not popular in Bangladesh. Moreover, I think there can’t be anything so impactful as face-to- health services. |
| 22 | I trust it. Very talented and efficient doctors work here. |
| 23 | In Bangladesh it is not possible for people sometimes to go to the doctors when needed. At that time, it can help us |
| 24 | From a practical point of view, it needs more engagement to the rural areas. I am extremely hopeful. |
| 25 | Highly expected. Because it will be a great innovation for Bangladesh. |
| 26 | Reproductive health care is important for preventing maternal and newborn deaths. It needs to be nurtured and protected throughout our lives, and we should all practice sexual self-care. |

# Q3

| **Sl. No** | **B3. How do you think that the face-to-face experiences of sexual and reproductive health services in Bangladesh influence young people's attitude and actual usage of reproductive mobile health services? Please give us an example from your life or others.** |
| --- | --- |
| 1 | Rather than digitalizing it and experiencing it, one must study academically about the reproductive health and system , that includes reproductive related education in schools and high schools. But in case of experiencing, its people become aware and feel the need to know about it more and then comes the mHealth . |
| 2 | Most of the time the experiences of sexual and reproductive health services are bad as still now these topics are considered as taboo to talk about, also the conservative mindset influences young people's attitude thus the reproductive mobile health service is more reliable, as it secure secrecy and nonjudgmental. |
| 3 | I have little idea I never discussed about it with young people. I never reach out people. |
| 4 | Might be positive. |
| 5 | It influences people in both negative and positive way. For example, someone may notice unusual symptom and seek doctor for further health checkup. Also, some may get paranoid over small changes and symptoms. That can make them anxious and stressed. Like period date may not synchronize with the app always. |
| 6 | Can’t think of any examples right now. |
| 7 | Positive correlations. |
| 8 | Girls are generally shy to share someone about their period, or personal health for this problem teenager are influenced to use reproductive mobile health service. |
| 9 | Sorry. No idea. |
| 10 | Can't tell. |
| 11 | Face to face experience often turns out to be fruitless since the doctors and healthcare professionals aren't always on par with the latest medical advances. And can't provide accurate treatments on the first go. Taken from a friend's experience. Which made her book a virtual appointment with a doctor from abroad. |
| 12 | Sexual and reproductive health issues are taken to be a taboo subject in our country and people generally do not talk about this openly, hence younger people remain unaware of these issues. Even such health services are also not commonly sought by most people. These mobile health services can promote these issues as well as make the younger generation aware about it too. Also, by using them, they can receive benefits of keeping track of their health issues. |
| 13 | Not experienced yet. |
| 14 | It's very effective nowadays as my mom get some anxiety issue because of her cancer treatments our family doctor suggested us to check her up a psychology as my mom was very sick couldn't move her anywhere .we took a psychiatrist appointment on online, and the doctor share a mental health app which quite helped to recover my mom. |
| 15 | Yes. |
| 16 | I don't have any experience yet. |
| 17 | I don’t have any example. |
| 18 | Face to face services may damage a person's mental health as in that kind of setting a service seeker is humiliated and often faces a traumatic experience which can lead her/him to use mobile or e-services. I don't have any examples. Personally, I have not used that kind of service. |
| 19 | Maybe in some cases. |
| 20 | Many of them are not comfortable in face-to-face experiences. |
| 21 | It will be beneficiary for the young people as they can get easy information and tips from here. But it’s not possible to understand the health issues without being consulted with a doctor. |
| 22 | Yes, it’s important to make the young generation conscious. |
| 23 | When i went to a doctor after my marriage, she told me about the total procedure of reproduction and eventually this knowledge helped us to lead a safe conjugal life. |
| 24 | I am not very sure about face-to-face experience regarding this service. But certainly, it will bring immense help. |
| 25 | Have no experience. |
| 26 | It protects both the mother and the child from infectious diseases and delivers a healthy baby. It provides complete knowledge about the early pregnancy, infertility, birth control methods, pregnancy, post-childbirth care of the baby and mother, etc. |

# Q4

| **Sl. No** | **B4. What do you think influences or might influence your intention to use reproductive mHealth services and how does it influence you? For examples, peers, social media, TV/Web advertisement, Automated SMS etc. might be a source of influence, how they influence you.** |
| --- | --- |
| 1 | Role of peers and social media. |
| 2 | Role of peers and social media. |
| 3 | Suggestion from friend and frequent web browsing. |
| 4 | Role of web advertisement. |
| 5 | Social media and peers influence most. |
| 6 | Friends, social media, YouTube, newspaper. |
| 7 | Role of social media. |
| 8 | Social media, friends. |
| 9 | Social media and advertisements. |
| 10 | Advertisement. |
| 11 | I think peers, television shows, advertisements on the internet might have a role here. |
| 12 | I got to know about these apps from YouTube ads basically. Then I talked to one of my seniors from university and tried a few apps myself till I found my preferred one. |
| 13 | Social media and advertisement sometimes influence me. |
| 14 | Yes, they do. By the TV/web I get some key information how to live happily how to get peace of mind. |
| 15 | It will influence people if they are taught the importance of it as a part of their regular health. |
| 16 | I can Learn many things from those which I didn’t know before. |
| 17 | Advertising through social media. Because it makes the service visible and mostly, we use social media. |
| 18 | Social media, as I get to know about people's experience of visiting gynecologists in Bangladesh. Most of the experiences were negative. |
| 19 | I didn’t influence by it. |
| 20 | Yes, they influence me. Because advertisements always influence me. social media is the first way to influence anyone in this century. |
| 21 | Social media, advertisements and automated SMS service can be a good source to get information. For example, during covid period, I got a lot of information through social media. |
| 22 | SMS, advertising. |
| 23 | Through various sources of media, we get alert sometimes like why irregular period happens, what will be its dangerous effect, how we can overcome it etc. |
| 24 | Social media potentially plays the role improve health outcomes, develop a professional network, increase personal awareness of news and discoveries, motivate patients, and provide health information to the community. |
| 25 | Advertisement and Automated SMS. |
| 26 | Community- and society-level factors influencing reproductive health include access to health care, immunizations, environmental quality, and low rates of injury and violence. |

# Q5

| **Sl. No** | **B5. How do you think self-awareness of one’s sexual and reproductive health (or overall health consciousness) influence their attitude, adoption and usage of mobile health services in Bangladesh? Please give us a personal story.** |
| --- | --- |
| 1 | It is needed to be aware about every single sexually transmittable diseases and reproductive health and to do that a step can be mobile health services. |
| 2 | Self-awareness affects to influence one's sexual and reproductive health as without awareness one will not Know the benefits and possibilities of Mobile health services in Bangladesh. |
| 3 | I believe that it is more because of anxiety, shyness and social stigma than self-awareness that people turn to mHealth service. Here awareness about mobile health services is todays networking it no wonder everyone uses mobile phone and internet people easily get to know about mobile health services but why they prefer to use it because they believe that they would not have to give away their identity. |
| 4 | Self-awareness might increase the usage of these services. |
| 5 | I do not know. |
| 6 | Sorry the question is not clear to me. |
| 7 | No comment. |
| 8 | Self-awareness about sexual and reproductive health is now increasing. |
| 9 | No such personal story. |
| 10 | No story. |
| 11 | Personally, being educated on your body is a huge plus and helps you gain confidence and rule out any superstitions that might come in the way. |
| 12 | I was facing hormonal changes and missed period for two consecutive months. Then I talked to a gynecologist and started following her advice. I basically started to use the apps to keep my period routines and symptoms. When i used to keep the tracks of my period manually, i often mixed up the dates but with the help of the app, now i get a journal of my period and overall health journey for the last 3 years. |
| 13 | As am not experienced yet I have no story about this. |
| 14 | By knowing other victims’ story can relate sometimes and how they survived how they handle the situation by knowing that get some courage to deal with this kind of situation but never faced any kind of situation personally. |
| 15 | Yes, of course. Can’t recall any story right now. |
| 16 | Mobile app service can provide many reliable information. |
| 17 | I don't have any story. But obviously when people are careful about their health, they tend to use Mobile health services more often. |
| 18 | Most of the people get sex education from pornography and they are often not aware of overall sexual health and reproductive health. Which can be detrimental. But the attitude, adoption and usage can be mostly visible if proper sex education is provided in a social setting. And awareness is increased. I still don't think this kind of service is widely used as it should be. Most of the people are shy, unaware and find it as taboo. |
| 19 | I don’t have any related story. |
| 20 | Sexual and reproductive health is a sensitive issue for both men and women. So to take care of sexual health self-awareness should be the first thing. |
| 21 | mHealth service will be helpful to spread health information and basic medical tips. But a major part of population in Bangladesh are uneducated. I wonder how they will manage. And if the get the misinformation or wrong medical services, it should be dangerous. |
| 22 | Yes, it influences. |
| 23 | I was suffering from irregular period since my childhood. After becoming adult I looked for information about why it happens and how I can overcome it. This self-awareness always forces me to be alert about this disease. |
| 24 | Self-awareness is the key factor for good sexual and reproductive health. From socio economic and cultural perspective of Bangladesh mobile heath service is very crucial.It allows me to talk to my partner about out sexual relationships, contraception etc. |
| 25 | Have no experience. |
| 26 | Sexuality education has positive effects, including increasing young people's knowledge and improving their attitudes related to sexual and reproductive health and behaviors. Sexuality education – in or out of schools – does not increase sexual activity, sexual risk-taking behavior or STI/HIV infection rates. |

# Q6

| **Sl. No** | **B6. At university level where students are more or less matured, what is the role of sexual and reproductive health knowledge, and technological knowledge shaping the attitude of students regarding mHealth services and how do you think it affects reproductive mHealth adoption and usage among female students?** |
| --- | --- |
| 1 | Technology makes everything easier, and it is a modern way of gaining knowledge. So, if that includes the reproductive related knowledge then it would be easier for everyone to gain basic and detailed information on it. |
| 2 | The role of both sexual and reproductive health and technological knowledge is massive for shaping the attitude of student's regarding mHealth as it helps the adaption and usage. |
| 3 | University students quickly learn and adopt using technology, especially smart phone. They use it like normal day tool like cooking utensils or dresses or any other appliances. So, I do not think any special knowledge is needed or related to develop an attitude (positive or negative) towards mobile health services. Evidence that a certain section student is technologically literate but not into using mobile health services even though using laptop mobile or even more advanced technology i don’t k ow why. |
| 4 | Technological knowledge might positively affect the usage. |
| 5 | I do not know. |
| 6 | It’s really important because the university students are grown up and they must know about their SRH, there must be campaigns in the university to break this taboo and to use mHealth. |
| 7 | Yes, I think. |
| 8 | Technological knowledge affects reproductive mHealth adoption and usage. because of it services according to personal health is can be easier day by day. |
| 9 | If awareness increases, then mHealth adoption and usage will also increase. |
| 10 | I have not seen any impact yet. |
| 11 | Mobile health services help you to understand your body and health at a primary level. Since having adequate sexual and reproductive health knowledge can increase students' awareness of the importance of seeking and utilizing health services, including reproductive health services. This knowledge can also help students understand the benefits of using mHealth services, such as the convenience and privacy they offer. |
| 12 | Very much. These apps promote and provide knowledge about sexual and reproductive health issues and one can learn a lot from them. Once one tries one of the services, I think they will surely keep on using them. |
| 13 | As most of the university students are unmarried so they are less thinking about their reproductive health and I found this from surroundings like senior, junior or batchmate. |
| 14 | Because of so many technologies there is a lot good and bed effects in our student’s life as at university level we started exploring so much that we don't have any boundaries as we easily get everything through technology. |
| 15 | It may help them to be open up with their problems related to reproductive health. And as they are already possessing the tertiary level education, they have much more opportunity to adopt and use those mHealth related services. Besides, they can work together to make people aware of this who are marginalized or less privileged in our society as a part of voluntary activities. |
| 16 | Very significant role they are playing in our life as it is a very sensitive issue. |
| 17 | Actually, when we have girls talk, we talk about current different diseases becoming continuous story we feel to be more careful and mobile service is more affordable via as it saves time. |
| 18 | I think target beneficiaries should not be university students. Rather they should be teenagers. |
| 19 | Yes, sometimes. |
| 20 | It’s more important for female students because many of them are not comfortable to share their problem with anyone because of taboo or shyness. They don’t want face to face experiences with doctor or anyone else regarding this issue. |
| 21 | At University level, female students are not only matured but also educated. So this will be useful to them. Because everyone will have easy access to services and it’s not costly. |
| 22 | Every student should have this knowledge. |
| 23 | In Bangladesh, at university level many female students enter into their sexual life. Then obviously there has a big role of sexual knowledge shaping the attitude of the students and it should be. |
| 24 | Bangladeshi female students are socially and culturally not very outspoken regarding sexual and reproductive health knowledge. It can help them to know age-appropriate and phased information covering human rights, gender equality, relationships, reproduction, dangerous behaviors and disease prevention |
| 25 | Yes. |
| 26 | Good sexual and reproductive health is important for women's general health and wellbeing. It is central to their ability to make choices and decisions about their lives, including when, or whether, to consider having children. |

# Q7

| **Sl. No** | **B7. What is your willingness of use or continue using mobile health services and willingness to recommend it to your friends and family? and why? Do you find any challenges?** |
| --- | --- |
| 1 | I have found significant benefits using mobile health services and gained awareness, so I will definitely suggest my relatives and friends to use these services as well. |
| 2 | My willingness to use or continue using mobile health services are it's easy, no cost and sustainable. I recommended it to my friends and family because I was benefitted by it so they should. |
| 3 | I still cannot fully resort to mobile health services. I may not surely say that I will recommend my friends or relatives. But yes, i will also not discourage if people start taking help from it and are genuinely helped from mobile health services. |
| 4 | I am somewhat willing to use these services. I will also recommend it because it may help my friends and family. |
| 5 | I'll recommend my family and friends to use mHealth services so that they can know what is good to eat, when, what symptoms are normal and what is alarming etc. |
| 6 | 5/5. As we are shy to discuss it face to face, it’ll be easier for us to talk about it over the mobile or any apps. I’ll also recommend it to my friends and family for their SRH and well-being. |
| 7 | Yes, I recommended. |
| 8 | Yes, I will be recommending it to my female friends and my younger sister and relative for that they know about the actual solution, and they can use it about their health and safety. |
| 9 | Willing. It feels affordable and very effective. So, I will recommend others. |
| 10 | might use if proved good enough for my health and will also recommend. |
| 11 | I do and will use several mHealth applications and often recommend them to others. Since it makes us health cautious, tracks menstrual cycle, calorie intake and usage, etc. And many other things that usually may slip our mind. Having an app do the heavy lifting is pretty useful. |
| 12 | I will always recommend every girl to try the apps once and find a suitable one for themselves. |
| 13 | I am willing to use mobile health services and if I will find this trustworthy and benefited from it in that case, I will suggest others to use it. |
| 14 | It helps us in various ways. Yes, I will recommend it because it will help them in a good way to leading a good mHealth. |
| 15 | Yes, I prefer. |
| 16 | I think it is very helpful specially for the teenage and by using those app they Don't need to feel shy to ask elders and i didn’t face any challenges while using these apps. |
| 17 | I am very much fond of using mHealth service and I encourage my friends and family to use it because it is time saving and takes little effort to keep us safe and advance a little. |
| 18 | I think the waiting period to get an answer can be long. And often the websites and apps are not updated. |
| 19 | Yes of course, if they need. No challenges. |
| 20 | Yes, I will continue using this service and will recommend it to my friends and family. There might be some challenges like some friend or family member might get me wrong while sharing this because of the social barriers of not sharing this health problems with anyone. |
| 21 | As I said before, it is useful in certain circumstances. I would recommend this but in major cases people must consult to doctor before applying it to their health. |
| 22 | I want to use this service. Working person remains busy. We Don't get much time to visit doctors. So it is needed for me. |
| 23 | We should use it as we get information more quickly and can take steps before consulting and going to a doctor. |
| 24 | I am looking forward to it. |
| 25 | No. |
| 26 | It creates awareness among adolescents about safe sexual practices. It helps in preventing sexually transmitted infections, including HIV/AIDS. It protects both the mother and the child from infectious diseases and delivers a healthy baby. It provides complete knowledge about the early pregnancy, infertility, birth control methods, pregnancy, post-childbirth care of the baby and mother, etc. There is limited challenges in mHealth services. |
