# Supplementary material for: Towards women’s digital health equity: A qualitative inquiry into attitude and adoption of reproductive mHealth services in Bangladesh
Source: PLOS Digit Health. 2024 Oct 15;3(10):e0000637. doi: 10.1371/journal.pdig.0000637 (PMC11478865; doi:10.1371/journal.pdig.0000637)
Supplement: Attachment — Submitted filename: Article Review-PDIG-D-24-00267.pdf [file pdig.0000637.s002.pdf]

Towards womenʹs digital health equity: A qualitative inquiry into attitude and adoption of reproductive mHealth services in Bangladesh- Review

A. SUMMARY OF THE RESEARCH FINDINGS AND OVERALL IMPRESSION

i. Summary of the Research findings

Abstract section

The authors have provided a background on the healthy equity in Bangladesh with regards to women's attitudes and behaviors concerning digital health. 26 women were interviewed, and the methodology used is clear "the Integrative Model of Planned Behavior (IMPB)." The results have clearly been presented; However, there are no specific demographic details of the women interviewed (yet key words talk about young women), the summary information on challenges faced in accessing reproductive health services is limited, the summary of policy implications and study limitations is missing, perceptions into barriers to adoption and specific recommendations from the authors are also missing. I understand this is a qualitative study, but surely, the quantitative data on adoption and impact, challenges in implementation is missing. Wouldn't this make better the arguments, what about the fact that are there specific app features that formed the discussions or conclusions? The authors talk about healthcare providers and policy makers but their perspectives are missing. How would they then benefit from this work when they are not involved? At the beginning of the abstract, the authors talk about socio-economic conditions and location, what about the socio-cultural factors (for example, somewhere on line 374, the authors mention that "the women and girls were reluctant to share their menstruation and reproductive health problems due to socio-cultural factors and stigmatization in
Society"; these seem to contribute a lot the results)? Lastly, the future research directions and policy recommendations for enhancing mHealth effectiveness in reproductive healthcare in Bangladesh is missing. And this is critical for this research. Since the abstract is a central place for reviewing the whole article, a summary of this information is also important to enable the readers have a one-time view of the article before digging down to the details.

Other sections

There is a good introduction clearly discussing the societal taboos surrounding reproductive health in Bangladesh and the potential of mHealth to empower women in making informed health decisions. the authors have stressed the importance of health equity in Bangladesh, particularly focusing on the disparities faced by different groups due to socio-economic conditions and geographical locations. Issues such as the role of digital health solutions, especially mHealth, in bridging these gaps and improving access to healthcare services, with a specific emphasis on reproductive health services for women have been well addressed.

The transformative potential of mobile health technologies in improving healthcare delivery and promoting health outcomes in Bangladesh has been discussed bringing out issues such as the convenience, accessibility, and privacy that mHealth services offer, emphasizing their

positive impact on awareness, diagnosis, prevention, and control of illnesses. The importance of understanding gender dynamics in digital health programs to enhance health outcomes has also been emphasized.

The methodology used is clear where a qualitative approach was used to investigate the women's behavioral intentions towards reproductive mHealth services, the selection of participants from diverse backgrounds, and the data collection and analysis processes.

However, detailed information on the specific criteria used for selecting the study participants, such as age range, educational background, and geographic diversity is missing. This could actually provide a clearer understanding of the sample characteristics and potential biases therein.

In addition to that, there is a gap in explaining the specific procedures followed during data collection, including the interview structure, duration, and any standardized tools used. I believe this could also impact the reliability and validity of the findings.

The number of women interviewed is really and it may not be so realistic to make conclusions for the entire Bangladesh based on only 26 women.

ii. Overall impression.

The authors have an impressive research, which explores women's access to reproductive health services in Bangladesh through digital health solutions. They have emphasized the importance of understanding attitudes and adoption behaviors towards mHealth services. The research highlights the potential of mobile health technologies in bridging healthcare disparities, especially for marginalized groups. The study also advocates for tailored services to enhance women's health equity in the country and discusses the challenges and opportunities of integrating mHealth into reproductive healthcare services in Bangladesh.

B. SPECIFIC AREAS OF IMPROVEMENT

- ✓ Add summary missing information in the abstract (and the details in the main document)
- ✓ Provide detailed information on the specific criteria used for selecting the study participants, such as age range, educational background, and geographic diversity
- ✓ Provide a brief explanation to readers on the specific procedures followed during data collection, including the interview structure, duration, and any standardized tools used.
- ✓ Provide a brief about the future research directions and policy recommendations for enhancing mHealth effectiveness in reproductive healthcare in Bangladesh.
- ✓ Would be interesting to include the perspectives of the healthcare providers and policy makers
