# Supplementary material for: Towards women’s digital health equity: A qualitative inquiry into attitude and adoption of reproductive mHealth services in Bangladesh
Source: PLOS Digit Health. 2024 Oct 15;3(10):e0000637. doi: 10.1371/journal.pdig.0000637 (PMC11478865; doi:10.1371/journal.pdig.0000637)
Supplement: Attachment — Submitted filename: Response to Reviewers.docx [file pdig.0000637.s003.docx]

**Response to Reviewers: *[Marked Yellow]***

Reviewer's Responses to Questions

**Comments to the Author**

1. Does this manuscript meet PLOS Digital Health’s [publication criteria](https://journals.plos.org/digitalhealth/s/journal-information#loc-criteria-for-publication)? Is the manuscript technically sound, and do the data support the conclusions? The manuscript must describe methodologically and ethically rigorous research with conclusions that are appropriately drawn based on the data presented.


Reviewer #1: Yes

Author’s Response: N/A

Reviewer #2: Yes

Author’s Response: N/A

Reviewer #3: Partly

Author’s Response: Authors believe that following revision of the manuscript, all criteria for publication were duly met.

--------------------

2. Has the statistical analysis been performed appropriately and rigorously?

Reviewer #1: Yes

Reviewer #2: Yes

Reviewer #3: N/A
Author’s Response: N/A

--------------------

3. Have the authors made all data underlying the findings in their manuscript fully available (please refer to the Data Availability Statement at the start of the manuscript PDF file)?

The [PLOS Data policy](https://journals.plos.org/digitalhealth/s/data-availability) requires authors to make all data underlying the findings described in their manuscript fully available without restriction, with rare exception. The data should be provided as part of the manuscript or its supporting information, or deposited to a public repository. For example, in addition to summary statistics, the data points behind means, medians and variance measures should be available. If there are restrictions on publicly sharing data—e.g. participant privacy or use of data from a third party—those must be specified.

Reviewer #1: Yes

Author’s Response: N/A

Reviewer #2: Yes

Author’s Response: N/A

Reviewer #3: No

Author’s Response: Regarding availability of transcripts of interviews, it was declared to be kept secret for the sake of participants identity. However, the methodology section outlined this issue even if the data is not currently made available. As part of qualitative research, anonymized final interview data can be shared if requested.

--------------------

4. Is the manuscript presented in an intelligible fashion and written in standard English?

PLOS Digital Health does not copyedit accepted manuscripts, so the language in submitted articles must be clear, correct, and unambiguous. Any typographical or grammatical errors should be corrected at revision, so please note any specific errors here.

Reviewer #1: Yes

Reviewer #2: Yes

Reviewer #3: Yes

Author’s Response: N/A

--------------------

**5. Review Comments to the Author**

Please use the space provided to explain your answers to the questions above. You may also include additional comments for the author, including concerns about dual publication, research ethics, or publication ethics. (Please upload your review as an attachment if it exceeds 20,000 characters)

**Reviewer #1:**

• The manuscript provides invaluable insights into women's attitudes towards reproductive health services in Bangladesh, a topic of urgent concern. It fills a crucial gap in the literature and underscores the potential impact of this research, offering hope for significant improvements in women's reproductive health.

• The Integrative Model of Planned Behavior (IMPB) is not just well-justified, but its application is also reassuringly appropriate, enhancing the credibility of the research.

• The findings highlight and illuminate essential themes such as the convenience and security of mHealth services, trust issues, and socio-cultural barriers.

Thanks for the meticulous observations regarding paper’s effort to contribute to reproductive health services in Bangladesh where such healthcare are poorly designed and fail to meet women’s expectations. We appreciate the compliments and believe this paper will help future policy makers, practitioners, service providers and researchers alike. Considering multitude of factors affecting women’s reproductive health, IMPB was selected for this research. Nonetheless, further justification behind choosing this model was added in the revised manuscript. [Line 190-194]

• The sample size is small and limited to university students, which may not fully represent the diverse experiences of women across Bangladesh. Future research should include a more varied demographic.

Author’s Response: Due to funding issues, we have only focused on the university students. Also, the current market of mHealth services is dominated by young and educated consumers which is also provided a feasible sample for this study. This logic was added to revised manuscript [Line 247-248; 231-235]. But of course, in future research we will surely follow the observed suggestion as we believe there is a huge potential to conduct a quantitative study based on the findings of this study that would involve more samples from across the country. [Line 528-532]

• More detail on ensuring the reliability and validity of the findings would strengthen the study.

Author’s Response: We have made changes according to the comment. [Line 257-265]

• Specific, actionable recommendations for policymakers and healthcare providers are beneficial and crucial for enhancing the study's practical utility and improving women's reproductive health in Bangladesh.

Author’s Response: The authors believe that in order to develop specific, actionable recommendations for policymakers and healthcare providers, it is imperative to obtain a more comprehensive and multi-stakeholder perspective regarding this issue. Still some observations were added to the conclusion . [Line 503-504; 516-518]

• The manuscript could benefit from a more critical discussion of the findings and their implications.

Author’s Response: Some revisions were added in the discussion part and implications in the conclusion.

**Reviewer #2:**

The analysis was done as required and the manuscript is presented in an acceptable fashion. what can be improved is adding an explanation on the ethical considerations beyond obtaining consent, such as ensuring confidentiality, handling sensitive information, and addressing any potential risks or conflicts of interest, which are crucial aspects in qualitative research involving human subjects.

Author’s Response: We have added more details regarding it to the manuscript. [line 283-290]

**Reviewer #3:**

Despite being a significant topic, the major concern associated with the submitted manuscript is the lack of proper sample size selection. Only 26 cases are not sufficient to provide any sort of interpretation. Additionally, the study seems to be more on review based study than the original research, so it could be applied as a review paper and not an original research.

Author’s Response: We strongly disagree with you on this matter. Public health research on women’s reproductive health is a sensitive issue and still taboo in the global South. Even 5–10 cases could be a significant number in such a situation. Nevertheless, the consensus among scholars is that the range of sample size most commonly observed in qualitative research is between 20 and 60. The foundation and structure of this research rely extensively on primary data collected from women. That is the reason why we chose to submit it to the section for original research instead of submitting it as a review paper.

--------------------

6. PLOS authors have the option to publish the peer review history of their article ([what does this mean?](https://journals.plos.org/digitalhealth/s/editorial-and-peer-review-process#loc-peer-review-history)). If published, this will include your full peer review and any attached files.

**Do you want your identity to be public for this peer review?** If you choose “no”, your identity will remain anonymous but your review may still be made public.

For information about this choice, including consent withdrawal, please see our [Privacy Policy](https://www.plos.org/privacy-policy).


Reviewer #1: No

Reviewer #2: No

Reviewer #3: No

Author’s Response: N/A

--------------------

__________________________________________________

**Editor’s Comments**

**A. SUMMARY OF THE RESEARCH FINDINGS AND OVERALL IMPRESSION**

**i. Summary of the Research findings**

**Abstract section**

The authors have provided a background on the healthy equity in Bangladesh with regards to women’s attitudes and behaviors concerning digital health. 26 women were interviewed, and the methodology used is clear “the Integrative Model of Planned Behavior (IMPB).” The results have clearly been presented; However, there are no specific demographic details of the women interviewed (yet key words talk about young women), the summary information on challenges faced in accessing reproductive health services is limited, the summary of policy implications and study limitations is missing, perceptions into barriers to adoption and specific recommendations from the authors are also missing.

Author’s Response: Your suggestions here are clear and specific, which is greatly appreciated by authors. In the abstract, demographic details of participants, challenges, policy implication and limitations all were added in this revision. While authors understand the necessity of detailing some key information in the abstract, they also factored the word limits for this. So, the effort was to provide concise information within a compact abstract that can help the readership comprehend the essence of this research. [Line 19-22; 26-28; 32-49]

I understand this is a qualitative study, but surely, the quantitative data on adoption and impact, challenges in implementation is missing. Wouldn’t this make better the arguments, what about the fact that are there specific app features that formed the discussions or conclusions? The authors talk about healthcare providers and policy makers but their perspectives are missing. How would they then benefit from this work when they are not involved?

Author’s Response: Yes, authors believe that quantitative data and the involvement of certain stakeholders would have helped to make a stronger case. But due to the design of this study as well as the lack of available funds, it was improbable to execute these suggestions. However, it has been added as a limitation for the study and authors hope this issue will be resolved in the future studies regarding such topic in Bangladesh. [Line 44-46; 524-526]

At the beginning of the abstract, the authors talk about socio-economic conditions and location, what about the socio-cultural factors (for example, somewhere on line 374, the authors mention that “the women and girls were reluctant to share their menstruation and reproductive health problems due to socio-cultural factors and stigmatization in Society”; these seem to contribute a lot the results)?

Author’s Response: Yes, as sharply pointed out, those socio-cultural factors definitely impact the reproductive health seeking behaviors, this was added in the revised abstract. [Line 15-16; 35-36]

Lastly, the future research directions and policy recommendations for enhancing mHealth effectiveness in reproductive healthcare in Bangladesh is missing. And this is critical for this research. Since the abstract is a central place for reviewing the whole article, a summary of this information is also important to enable the readers have a one-time view of the article before digging down to the details.

Author’s Response: Thanks for pointing that out, it was incorporated briefly in the revised abstract. [Line 40-44]

**Other sections**

There is a good introduction clearly discussing the societal taboos surrounding reproductive health in Bangladesh and the potential of mHealth to empower women in making informed health decisions. the authors have stressed the importance of health equity in Bangladesh, particularly focusing on the disparities faced by different groups due to socio-economic conditions and geographical locations. Issues such as the role of digital health solutions, especially mHealth, in bridging these gaps and improving access to healthcare services, with a specific emphasis on reproductive health services for women have been well addressed. The transformative potential of mobile health technologies in improving healthcare delivery and promoting health outcomes in Bangladesh has been discussed bringing out issues such as the convenience, accessibility, and privacy that mHealth services offer, emphasizing their positive impact on awareness, diagnosis, prevention, and control of illnesses. The importance of understanding gender dynamics in digital health programs to enhance health outcomes has also been emphasized.

Author’s Response: Thanks for the thorough observation. The main objective of the paper was, as mentioned in the title, about the health equity especially at the age of digitalization of healthcare. As a little explore topic in the context of a developing country like Bangladesh, this study intended to fill both empirical and literature gap regarding digital health equity. We believe that this study can help future research and policy targeted women’s health equity in developing countries.

The methodology used is clear where a qualitative approach was used to investigate the women's behavioral intentions towards reproductive mHealth services, the selection of participants from diverse backgrounds, and the data collection and analysis processes. However, detailed information on the specific criteria used for selecting the study participants, such as age range, educational background, and geographic diversity is missing. This could actually provide a clearer understanding of the sample characteristics and potential biases therein.

Author’s Response: Authors tried to present a detailed picture of how the study was carried out and also ensure the saturation of data free from potential biases. Though the sample was drawn from young and educated population because they were likely be the among first to use or being exposed to digital health services in Bangladesh where digital literacy is low. Based on the suggestions above, more information about selection criteria were added in this revision of the manuscript along with the reasonings. [Line 231-238; 247-248]

In addition to that, there is a gap in explaining the specific procedures followed during data collection, including the interview structure, duration, and any standardized tools used. I believe this could also impact the reliability and validity of the findings. The number of women interviewed is really and it may not be so realistic to make conclusions for the entire Bangladesh based on only 26 women.

Author’s Response: Thanks for pointing out the lack of details in the procedures which have been addressed in the current revision. Especially the three pilot interviews and specifics of interview procedures were added. As indicated in the study our study reached a saturation point after 26 interviews and we believed that it was enough to make some conclusions because other section of the population could not have the same level of the digital literacy as young and educated people in Bangladesh. However, it is possible that inclusion of more samples would help us make the case stronger, also this limitation was already addressed in the conclusion. [Line 257-265; 524-527]

**ii. Overall impression.**

The authors have an impressive research, which explores women's access to reproductive health services in Bangladesh through digital health solutions. They have emphasized the importance of understanding attitudes and adoption behaviors towards mHealth services. The research highlights the potential of mobile health technologies in bridging healthcare disparities, especially for marginalized groups. The study also advocates for tailored services to enhance women's health equity in the country and discusses the challenges and opportunities of integrating mHealth into reproductive healthcare services in Bangladesh.

Author’s Response: Thank you for the kind words.

**B. SPECIFIC AREAS OF IMPROVEMENT**

- Add summary missing information in the abstract (and the details in the main document)

Author’s Response: The suggestion made above regarding the abstract has been duly incorporated in the revised manuscript. [Line 15-49]

- Provide detailed information on the specific criteria used for selecting the study participants, such as age range, educational background, and geographic diversity

Author’s Response: More details were added about the specifics of participant selection especially age range, educational background, and attempts towards geographic diversity. These were discussed above in “***other sections***” of editor’s comments. [Line 231-238; 247-248]

- Provide a brief explanation to readers on the specific procedures followed during data collection, including the interview structure, duration, and any standardized tools used.

Author’s Response: Further details about the specific procedures in the data collection process were added based the editor’s observation and suggestion which were discussed above as well. [Line 257-265]

- Provide a brief about the future research directions and policy recommendations for enhancing mHealth effectiveness in reproductive healthcare in Bangladesh.

Author’s Response: Briefs about policy recommendation and future research direction were added in the conclusion section. We believe that while specific policy is necessary for regulation of mobile health services and devices, a quantitative focus of the mHealth effectiveness would greatly benefit customization and better services in reproductive healthcare. [Line 503-504; 516-518; 531-532]

- Would be interesting to include the perspectives of the healthcare providers and policy makers

Author’s Response: We also believe that would have been interesting but due to the lack of funding, which was not possible, though it was added from secondary sources in the second section. However, it has been acknowledged as a limitation of our study in the conclusion. [Line 524-526]
